# Relationship of Mental Health, Social Support, and Coping Styles among Graduate Students: Evidence from Chinese Universities

**Published:** 2018-05

**Authors:** Pengju WANG, Zhuang XIONG, Hua YANG

**Affiliations:** 1. Center of Systems and Industrial Engineering, Zhongyuan University of Technology, Zhengzhou, P.R. China; 2. School of Management, Wuhan University of Technology, Wuhan, P.R. China; 3. Zhongyuan-Petersburg Aviation College, Zhongyuan University of Technology, Zhengzhou, P.R. China

**Keywords:** Graduate students, Mental health, Social support, Coping style

## Abstract

**Background:**

The increasing number of graduate students in China has resulted in the wide concern for their mental health problems. The coping style and social support are important factors that affect the mental health of individuals. This study aims to explore the relationship of the mental health, social support, and coping style of graduate students.

**Methods:**

The sample consisted of 260 graduate students from three universities of China. The participants were evaluated using the Symptom Checklist-90 (SCL-90), Social Support Revalued Scale (SSRS), and Coping Style Questionnaire (CSQ) in October and November 2017. The data of the scale were analyzed with *t*-test, correlation, and multiple regression analysis.

**Results:**

The graduate students had lower scores than the national norm standard on all subscales, except for anxiety and phobic anxiety in the SCL-90. Graduate students’ mental health was significantly negatively correlated with social support, problem-solving, and help-seeking and significantly positively correlated with self-blame, fantasy, withdrawal, and rationalization. Coping style and social support affected the mental health of graduate students, in which the regression coefficients of the subscales of problem-solving, rationalization, self-blame, and fantasy were −0.168, 0.070, 0.125, and 0.113, respectively. The regression coefficients of the subscales of subjective and objective supports were −0.086 and −0.024, respectively.

**Conclusion:**

The positive coping style and social support improve the level of graduate students’ mental health through the gain effect and alleviate abnormal psychological symptoms. The conclusions of this study can provide a reference to improve the psychological intervention strategies for graduate students.

## Introduction

The development of higher education in China has resulted in the increasing number of graduate students in Chinese universities. According to data from China’s National Bureau of Statistics (1), the number of graduate students in Chinese universities reached 1.981 million in 2016, up by 65.8% from 1.195 million in 2007. The expansion of graduate enrollment brings significant challenges to the graduate education and management of universities. Particularly, graduate students have profound pressure on economic, marital, academic, interpersonal, and employment concerns, and the high self-expectation and achievement motivation of graduate students are bound to affect their mental health. In recent years, the malignant crisis caused by mental health problems has occurred repeatedly. The results of related research corroborated that the rate of suicides of graduate students was higher than that of undergraduates (2). Psychological problems have become an important factor that affects the growth of graduate students and have aroused widespread concern in the society.

Moreover, coping style and social support were important mediating and moderating factors in the process of individual psychological stress, which exerted an important influence on the mental health of individuals and played a moderating role between stressors and stress reaction (3, 4). Generally, when encountering setbacks and pressures, individuals use their cognitive and behavioral ways to release nervousness, which could reduce the level of psychological stress (5). Social support, as individuals gain emotional, material, and life help from the social network, dissolves individuals’ psychological pressure (6). However, differences vary among the different groups of coping styles and social support, which are influenced by individual characteristics and external environmental factors. Therefore, graduate students have distinct characteristics in coping style and social support, which affect their mental health.

Given the background, this study seeks to answer, “What is the relationship of the mental health, social support, and coping styles of graduate students?” Graduate students from Chinese universities are the research objects of this study, which explored the relationship of their mental health, social support, and coping styles. Furthermore, this study explored and attempted to reveal the influence mechanism of coping style and social support on the mental health of graduate students. The conclusions were used to formulate and propose recommendations for psychological intervention strategies for graduate students.

### Literature review

Graduate studies encounter considerable psychological stress as a result of the significant differences with other student groups in age, experience, learning life style, pressure, and environment (7). However, the assessment of the mental health of the graduate students by survey tools, such as SCL-90, affirmed that the results of the related research were controversial and formed two different views on “bad theory” and “good theory” of graduate students’ mental health (8). Toews et al. (9) affirmed that the SCL-90 score of the graduate students was the highest and that subscale depression and anxiety scores were significantly higher than those of the other two groups. Cahir and Morris (10) assessed graduate students’ mental health and validated that the average SCL-90 score of the graduate students was higher than that of the non-patient standard. By comparing with the national norm, Liu et al. (11) verified that the overall mental health status of Chinese graduate students was lower than the average level of Chinese youth. The graduate students’ mental health status was pessimistic (12). Moreover, all subscale scores, except for somatization and interpersonal sensitivity, were significantly higher than those of the national norm.

However, other studies confirm the good trend of graduate students’ mental health. The level of graduate students’ mental health was similar to the norm of Chinese adults and that the overall situation was better than the average level of Chinese normal adults (13). Graduate students’ subscale somatization score of SCL-90 was significantly lower than that of undergraduate students with a comparative analysis of the mental health of graduate and undergraduate students (14). Dyrbye et al. (15) affirmed that the incidence of psychological problems was not significantly different from those of their peers through investigating medical graduates’ mental health. Mao et al. (16) reported no significant difference in the SCL-90 score of Chinese graduates and undergraduates. Most graduate students’ mental health score was close to that of Chinese normal adults.

In addition, existing research validated that many factors affected the mental health of graduate students. Personality traits were important predictors of mental health, especially neuroticism and psychoticism, which were highly correlated with mental health and could sufficiently explain the mental health variation (17). Graduation, employment, marriage, and cognitive skills were the main factors that influenced graduate students’ mental health (18). Huang et al. (19) proved that positive coping style of undergraduates was negatively correlated with all subscales of the SCL-90 and that the negative coping style was positively correlated with all subscales of the SCL-90. Differences in gender, professional knowledge, and other individual traits are the causes of significant differences in coping style (20). The lack of social support for college students led to psychological problems, especially for students who were different from most students (21). Family factors played an important role in individual psychological growth, self-control, and learning habits, which further affected the mental health of graduate students (22).

## Methods

### Research Tools

This study focused on graduate students’ mental health, and the relationship among mental health, social support, and coping style was analyzed. Therefore, we followed the principle of combining authority and applicability in the choice of research tools (23), and adopted authoritative scale, including Symptom Checklist-90, Coping Style Questionnaire, and Social Support Revalued Scale.

#### 1) Symptom Checklis-90

Symptom Checklis-90 (SCL-90) was compiled by Derogatis. SCL-90 has good reliability and validity and is widely used in the research of mental disorders and psychological diseases. SCL-90 consists of 90 items, uses a 5-point scale (1=“no problem” to 5=“very serious”), and includes 9 subscales, namely, somatization, obsessive–compulsive, interpersonal sensitivity, depression, anxiety, hostility, phobic anxiety, paranoid ideation, and psychoticism (24). SCL-90 is used to assess individual psychological symptoms, such as feeling, emotion, thinking, behavior, life habits, interpersonal relationships, diet, and sleep. Higher scores on the SCL-90 indicate profound psychological distress. When the total SCL-90 score is >160 or a subscale score is >2, respondents have abnormal psychological performance.

#### 2) Coping Style Questionnaire

The Chinese version of the Coping Style Questionnaire (CSQ) was compiled by Xiao et al. (25) in accordance with behavior habits of the Chinese people. The CSQ includes 62 items, divided into 6 subscales, namely, problem-solving, self-blame, help-seeking, fantasy, withdrawal, and rationalization, to measure the coping strategies of individuals in encountering stressful events. Respondents provide a “yes” (score=1) or “no” (score=0) response to each of the 62 items, with high scores reflecting a significant effect of the coping style on the respondent’s way of dealing with problems. CSQ has good reliability and validity, the factor load value of each subscale item is above 0.35, and the tool is widely used in localization research in China on coping style (26).

#### 3) Social Support Revalued Scale

The measurement of social support uses the Social Support Revalued Scale (SSRS) designed by Xiao (27). SSRS draws on the Social Support Questionnaire (SSQ) and Interview Schedule for Social Interaction (ISSI), and follows the principle of validity and simplicity. The 10 items are divided into 3 subscales, which are objective support (3 items), subjective support (4 items), and social support utilization (3 items). By the calculation of the sum of item scores in different subscales, the actual objective support, subjective experience, and individual utilization of social support are measured.

The evaluation results of the SCL-90, CSQ, and SSRS of the respondents were analyzed by using the SPSS 23.0 software. Correlation and regression analyses were used to explore the relationship among each subscale of the SCL-90, social support, and coping style.

### Data Sources

A random sampling method was adopted. In October and November 2017, a questionnaire survey was conducted among graduate students in three universities of China, which were Henan University of Technology, Zhongyuan University of Technology, and North China University of Water Resources and Electric Power. A total of 285 questionnaires were distributed, 269 were recovered, and the recovery rate was 94.4%. Nine invalid questionnaires were eliminated as these forms were filled out with incomplete and consistent answers. Finally, 260 valid questionnaires were obtained, with an effective recovery rate of 91.2%. Through the induction of effective questionnaire data, the descriptive statistics of the sample are shown in Table 1.

**Table 1:** Statistical description of sample

***Variable***	***Category***	***Frequency***	***Percentage***
Gender	Male	144	55.4
	Female	116	44.6
Grade	First grade	87	33.5
	Second grade	91	35.0
	Third grade	82	31.5
Whether the only child	The only child	155	59.6
	None-only children	105	40.4
Student source	Urban areas	117	45.0
	Rural areas	143	55.0
Student category	New graduates	140	53.8
	Past graduates	120	46.2

## Results

### Evaluation scores of graduate students’ mental health status, coping style, and social support

Tables 2, 3, and 4 exhibit the evaluation results of the mental health, coping style, and social support of the respondents according to the score calculation of SCL-90, CSQ, and SSRS, respectively.

**Table 2:** Graduate students’ mental health status

***Subscale***	***Score***	***Psychological abnormality***		***Normal standard***	***t-Test***
		***n***	***%***		
Somatization	1.29 ± 0.41	16	6.2	1.37 ± 0.48	−1.976
Obsessive–compulsive	1.42 ± 0.52	28	10.8	1.62 ± 0.58	−3.719^**^
Interpersonal sensitivity	1.37 ± 0.43	20	7.7	1.65 ± 0.61	−6.473^**^
Depression	1.38 ± 0.51	16	6.2	1.50 ± 0.59	−2.326^*^
Anxiety	1.42 ± 0.55	24	9.2	1.39 ± 0.43	0.547
Hostility	1.34 ± 0.42	20	7.7	1.46 ± 0.55	−2.840^**^
Phobic anxiety	1.29 ± 0.40	16	6.2	1.23 ± 0.41	1.397
Paranoid ideation	1.33 ± 0.54	24	9.2	1.43 ± 0.57	−1.868
Psychoticism	1.26 ± 0.39	8	3.1	1.29 ± 0.42	−0.692
Total score	121.32 ± 3.54	20	7.7		

* *P* < 0.05,

** *P* < 0.01

**Table 3:** Graduate students' coping style

***Subscale***	***Score***	***Male (n = 144)***	***Female (n = 116)***	***t-Test***
Problem-solving	0.72 ± 0.25	0.72 ± 0.20	0.72 ± 0.20	−0.129
Self-blame	0.34 ± 0.28	0.37 ± 0.25	0.31 ± 0.20	1.034
Help-seeking	0.57 ± 0.26	0.58 ± 0.21	0.55 ± 0.22	0.606
Fantasy	0.45 ± 0.24	0.48 ± 0.22	0.41 ± 0.16	1.352
Withdrawal	0.39 ± 0.23	0.41 ± 0.20	0.37 ± 0.17	0.718
Rationalization	0.44 ± 0.20	0.44 ± 0.17	0.45 ± 0.14	−0.217

**Table 4:** Social support for graduate students

***Subscale***	***Score***	***Male (n = 144)***	***Female (n = 116)***	***t-Test***
Objective support	10.31 ± 2.88	10.03 ± 2.82	10.66 ± 2.96	−0.872
Subjective support	20.68 ± 3.51	21.25 ± 3.18	19.97 ± 3.83	1.479^*^
Support utilization	7.71 ± 1.77	7.67 ± 1.82	7.76 ± 1.73	−0.207
Total score	38.69 ± 5.32	38.94 ± 4.59	38.38 ± 6.17	0.423

* *P* < 0.05

Table 2 shows that, on the basis of the SCL-90 subscale scores of the respondents, an increased number of prominent psychological abnormalities (subscale score >2) emerged. The subscales obsessive–compulsive, anxiety and paranoid ideation account for 10.8%, 9.2%, and 9.2%, respectively. The incidence rate of these psychological symptoms is approximately 10%, whereas those of somatization, depression, phobic anxiety, hostility, interpersonal sensitivity, and psychoticism are relatively low. In addition, the results of the survey are compared with the standard of the Chinese population norm. Each subscale score, except for anxiety and phobic anxiety, was lower than the national norm, whereas anxiety and phobic anxiety were slightly higher than the national norm. Moreover, obsessive–compulsive, interpersonal sensitivity, and hostility revealed significant differences compared with the national norm at the *P*< 0.01 level. The score for depression was significantly lower than the national norm at the *P* < 0.05 level. The incidence of psychological symptoms among graduate students in terms of obsessive– compulsive, interpersonal sensitivity, depression, and hostility was significantly lower than that of the national population norm.

Table 3 shows that the scores of male graduate students in the self-blame, help-seeking, fantasy, and withdrawal subscales were slightly higher than those of female graduate students. Furthermore, their combined scores in problem-solving and rationalization were basically the same. However, *t*-test affirms that a significant difference did not exist in the coping style between male and female graduate students. The result confirms that, in coping with stressful environment or events, gender differences were insignificant in terms of coping strategies to alleviate emotional problems.

Table 4 shows that the scores of male graduate students in objective support and support utilization were lower than those of female graduate students. However, male graduate students scored higher in the subjective support subscale than the female graduate students. Furthermore, significant gender differences existed in the subjective support subscale as revealed by the *t*-test. Male graduate students gained additional emotional experience, such as respect, support, and understanding, in their study life compared with the females.

### Correlation analysis of mental health, coping style, and social support

Table 5 presents the results of the correlation analysis among graduate students’ mental health, coping style, and social support.

**Table 5:** Coefficient of the correlation among graduate students’ mental health, coping style, and social support

***Variable***	***Problem-solving***	***Self-blame***	***Help-seeking***	***Fantasy***	***Withdrawal***	***Rationalization***	***Social support***
Somatization	−0.008^**^	0.171^**^	−0.184^*^	0.270^*^	0.218^*^	0.057^*^	−0.044^*^
Obsessive–compulsive	−0.321^**^	0.015^*^	−0.064^**^	0.039^**^	0.023^**^	0.068^**^	−0.225^**^
Interpersonal sensitivity	−0.138^**^	0.053^*^	−0.170^**^	0.040^**^	0.107^**^	0.050^*^	−0.058^**^
Depression	−0.125^**^	0.022^**^	−0.216^**^	0.098^**^	0.111^**^	0.052^*^	−0.129^**^
Anxiety	−0.063^**^	0.144^**^	−0.136^**^	0.205^**^	0.287^**^	0.140^**^	−0.134^*^
Hostility	−0.045^**^	0.105^**^	−0.036^**^	0.161^**^	0.137^*^	0.083^**^	−0.052^*^
Phobic anxiety	−0.039^**^	0.131^**^	−0.052^**^	0.215^**^	0.170^**^	0.080^**^	−0.013^*^
Paranoid ideation	−0.072^**^	0.019^**^	−0.183	0.118^*^	0.042^**^	0.023^**^	−0.046^**^
Psychoticism	−0.114^**^	0.036^*^	−0.200^**^	0.183^**^	0.149^**^	0.023^*^	−0.153^*^

* *P* < 0.05,

** *P* < 0.01

The CSQ scores for the subscales self-blame, withdrawal, fantasy, and rationalization were significantly positively correlated with all subscales of the SCL-90 (*P* < 0.05 or *P* < 0.01), which shows that under high stress, adopting immature or mixed coping styles cause graduate students’ psychological abnormality. A significantly negative correlation existed between the problem-solving subscale score and each subscale score of the SCL-90, which shows that graduate students could improve their mental health by applying reasonable and mature solutions to stressful events. However, the score for the help-seeking subscale was not significantly correlated with paranoid ideation but negatively correlated with other subscale scores of the SCL-90, which shows that the graduate students could effectively alleviate the abnormal psychological performance in high stress by means of help-seeking. In addition, social support had a significantly negative correlation with each subscale score of the SCL-90, which shows that the more the social support, the higher the utilization. Thus, the occurrence of graduate students’ psychological abnormality can be effectively reduced.

### Regression analysis of graduate students’ mental health, coping style, and social support

For the analysis of the factors that influence graduate students’ mental health, the SCL-90 total score was taken as the dependent variable and each subscale score of the CSQ and SSRS as independent variables for multiple regression analysis. Table 6 presents the results of the analysis. Problem-solving (*β* = −0.168) and rationalization (*β* = −0.070) had a significantly negative impact on mental health (*P* < 0.01). The subscales self-blame and fantasy had a significantly positive impact on mental health (*P* < 0.01). On the basis of the absolute degree of influence, the effect of problem-solving was high, followed by self-blame and fantasy, whereas rationalization had the least effect.

**Table 6:** Regression results of graduate students’ mental health, coping style, and social support

***Independent Variable***	***Normalized regression coefficient β***
Problem-solving	−0.168^**^
Self-blame	0.125^**^
Help-seeking	−0.238
Fantasy	0.113^**^
Withdrawal	0.084
Rationalization	0.070^**^
Objective support	−0.086^*^
Subjective support	−0.024^*^
Support utilization	−0.199
ΔR^2^	0.130
ΔF	25.910^**^

* *P* < 0.05,

** *P*< 0.01

The subscales objective support (*β* = −0.086) and subjective support of the SSRS (*β* = −0.024) had a significantly negative impact on mental health (*P*< 0.05), whereas the impact of support utilization was insignificant. The influencing factors of graduate students’ mental health include the subscales problem-solving, rationalization, self-blame, and fantasy of the CSQ, as well as the subscales objective support and subjective support of the SSRS.

## Discussion

The mental health of the 260 graduate students in this survey is good, and the symptom incidence of obsessive–compulsive, anxiety, and paranoid ideation is relatively high, at approximately 10%. Closely related to high levels of pressure, these psychological symptoms require tutors and management of universities to give highly value to these aspects. In addition, compared with the national norm, the incidence of psychological symptoms, such as obsessive–compulsive, interpersonal sensitivity, depression, and hostility, is significantly reduced. The reason for this phenomenon may be closely related to educational level. Graduate students have comprehensive knowledge structure and skills and master certain strategies and methods to cope with psychological stress during challenging events due to the long-term acceptance of higher education, and advantages such as personal knowledge stock and utilization. Results further affirm that the higher the level of education, the better that the individual can cope with psychological pressure. Therefore the negative states of burnout and depression caused by psychological pressure are inhibited. This result aligns with the findings of Zhi et al. (28). Therefore, in the face of a stressful environment or events, graduate students can alleviate their stress by rationally solving their problems and thus reduce the occurrence of psychological problems. The results of the investigation on coping style also confirm this finding. In Table 3, the scores of subscales problem-solving and help-seeking of the CSQ are significantly higher than those of other subscales. Hence, graduate students seldom adopt an immature coping style, such as withdrawal, fantasy, rationalization, and self-blame when encountering difficulties and pressure. In coping with cognition, graduate students can deal with stressful events, analyze problems rationally, and actively and pertinently seek solutions to problems. A significant gender difference does not exist in graduate students’ choice of coping style, which shows that, with the improvement of education, female graduate students are free from the influence of Chinese traditional culture on female stereotypes and role expectations.

The score for the subjective support subscale is obviously higher than that of the objective support and support utilization subscales. This result indicates that graduate students have a relatively high degree of emotional experience and satisfaction for being respected, supported, and understood in the current society. The reason for this is that graduate education is the top of educational chain, and the mode of education is commonly a one-on-one tutorial system. Therefore, in terms of study, tutors and university management departments tend to give increased attention and support to graduate students. Moreover, significant gender differences exist in the subjective support subscale. Male graduate students’ degree of subjective emotional experience is obviously higher than that of the females. The reason for this phenomenon may be that females are inclined to confide and ask for help when encountering problems, whereas males are highly independent, and their emotional expectation of support and understanding is easier to meet. In addition, the scores for objective support and support utilization are relatively low and form a big gap with subjective support. This gap exists because although the relevant management system at the present stage continually improves the living and academic support for graduate students, an effective transformation mechanism for objective material support is lacking. Thus, solving their problems through the support of these institutions is difficult, which can be further verified by score of the support utilization subscale (7.71 ± 1.77).

Graduate students’ mental health is significantly negatively correlated with the problem-solving and help-seeking subscales of the CSQ and significant positively correlated with the subscales self-blame, fantasy, withdrawal, and rationalization, but negatively correlated with the social support subscale, which is consistent with Li’s findings (29). Results validate that, when encountering stressful situations or events, graduate students who use mature coping styles, such as problem-solving and help-seeking, can effectively alleviate their abnormal psychological performance. Meanwhile, using immature or mixed coping styles, such as self-blame, fantasy, withdrawal, and rationalization, would aggravate the negative and bad mental state of graduate students. Improving graduate students’ mental health will further motivate them to adopt good coping styles during stressful events. In addition, increased social support can relieve the psychological pressure on graduate students and reduce the occurrence of psychological problems. Thus, a high level of mental health can obtained further social support.

The subscales problem-solving, rationalization, self-blame, and fantasy of the CSQ and subscales objective support and subjective support of the SSRS are the factors that influence graduate students’ mental health. The subscales problem-solving, objective support, and subjective support have a significantly negative impact on mental health, whereas the subscales rationalization, self-blame, and fantasy have a significantly positive impact on mental health. That is, objective support and subjective support should be strengthened by adopting positive coping styles to solve problems and improve the management system of graduate students in universities, which can reduce their negative psychology. Under high stress, graduate students who adopt immature or mixed coping styles, such as rationalization, self-blame, and fantasy, can effectively predict their mental health problems. In addition, ΔR^2^ = 0.13 (Table 6) implies that coping style and social support only explain 13% of the impact of mental health. In addition to coping style and social support, other factors also influence the mental health of graduate students. Therefore, psychological intervention for graduate students should not only guide them to adopt positive coping styles and give additional social support in encountering problems but also take more factors into consideration.

## Conclusion

The incidence rate of obsessive–compulsive, paranoid ideation, and anxiety of the SCL-90 is relatively high, whereas the other subscale scores of the SCL-90, except for anxiety and phobic anxiety, are lower than those of the Chinese population norm. Graduate students’ mental health is significantly negatively correlated with social support, problem-solving, and help-seeking of the CSQ, but has a significantly positive correlation with self-blame, fantasy, withdrawal, and rationalization of the CSQ. The problem-solving, rationalization, self-blame, and fantasy subscales of the CSQ, as well as the objective support and subjective support subscales of the SSRS, effectively predict graduate students’ mental health.

## Ethical considerations

Ethical issues (Including plagiarism, Informed Consent, misconduct, data fabrication and/or falsification, double publication and/or submission, redundancy, etc.) have been completely observed by the authors.

## References

[1] National Bureau of Statistics of China (2017). China Statistical Yearbook—2016. China Statistics Press, Beijing.

[2] Feng R Zhang YT Ma XT (2015). Analysis of research and progress in mental health education for graduate students in china in the past thirty years—based on review of CNKI literature between 1983 and 2013. J Grad Educ, 1: 21–5.

[3] Ando M (2002). Relationships among mental health, coping styles, and mood. Psychol Rep, 90(2): 606–12. 12061603 10.2466/pr0.2002.90.2.606

[4] Lakey B Orehek E (2011). Relational regulation theory: A new approach to explain the link between perceived social support and mental health. Psychol Rev, 118(3): 482–95. 21534704 10.1037/a0023477

[5] Wood AM Joseph S Linley PA (2007). Coping style as a psychological resource of grateful people. J Soc Clin Psychol, 26(9): 1076–93.

[6] Coyne JC Downey G (1991). Social factors and psychopathology: Stress, social support, and coping processes. Annu Rev Psychol, 42(1): 401–25. 2018399 10.1146/annurev.ps.42.020191.002153

[7] Hyun JK Quinn BC Madon T (2006). Graduate student mental health: Needs assessment and utilization of counseling services. J Coll Student Dev, 47(3): 247–66.

[8] Jiang S Huang Q (2016). A cross-temporal meta-analysis of changes in Chinese postgraduates’ mental health. J Nanjing Med Univ, 16(4):281–7.

[9] Toews JA Lockyer JM Dobson DJ (1993). Stress among residents, medical students, and graduate science (msc/phd) students. Acad Med, 68(10): S46–8. 8216630 10.1097/00001888-199310000-00042

[10] Cahir N Morris RD (1991). The psychology student stress questionnaire. J Clin Psychol, 47(3): 414–7. 2066411 10.1002/1097-4679(199105)47:3<414::aid-jclp2270470314>3.0.co;2-m

[11] Liu CY An JB Kang R (2005). Analysis on the psychological health and personality test of graduate students in Beijing. Chin J Behav Med Sci, 14(12):1121.

[12] Lou CL Lin LF Yuan XX (2003). Evaluation and analysis of the mental health status of graduate students. Explor Educ Dev, 12: 78–80.

[13] Li FY Ma Y Liu JW (2010). Survey of medical graduates’ mental health. Northwest Med Educ, 18(2): 284–7.

[14] Kontoangelos K Tsiori S Koundi K (2015). Greek college students and psychopathology: new insights. Int J Environ Res Public Health, 12(5): 4709–25. 25938913 10.3390/ijerph120504709 PMC4454935

[15] Dyrbye LN Thomas MR Shanafelt TD (2005). Medical student distress: causes, consequences, and proposed solutions. Mayo Clin Proc, 80(12): 1613–22. 16342655 10.4065/80.12.1613

[16] Mao FQ Li ZT Wang JH (2003). Psychosomatic symptom, personality factor and life event of graduate students. Chin Ment Health J, 17(10): 663–5.

[17] Lu L (1994). University transition: Major and minor life stressors, personality characteristics and mental health. Psychol Med, 24(1): 81–7. 8208897 10.1017/s0033291700026854

[18] Feng R Zhang Y (2017). A study on influential factors to psychological anxiety of doctoral students at school based on grounded theory. J Grad Educ, 3: 41–6.

[19] Huang W Zhou W Cheng QM (2006). Analysis on psychological health of college students in social support and dealing method. Chin J Public Health, 22(2): 139–40.

[20] Xiang Y Wang R Jiang Y (2016). Relationships among personality, coping style, and negative emotional response in earthquake survivors. Soc Behav Personal, 44(3): 499–507.

[21] Hefner J Eisenberg D (2009). Social support and mental health among college students. Am J Orthopsychiat, 79(4): 491–9. 20099940 10.1037/a0016918

[22] Strage AA (1998). Family context variables and the development of self-regulation in college students. Adolescence, 33(129): 17–32. 9583657

[23] Muthiah K Suja S (2017). A study on sense, feel, think, act, relate factors of experiential marketing in retailing. Transform Bus Econ, 16(1):85–99.

[24] Jurma AM (2015). Impact of divorce and mother’s psychological well-being on children’s emotional, behavioral, and social competences. Rev Cercet Interv So, 48:69–82.

[25] Xiao JH Xu XF (1996). Study on validity and reliability of coping style questionnaire. Chin Ment Health J, 4:164–8.

[26] Zhang M Lei KC (2002). Development of the coping style scale of undergraduates. Chin Ment Health J, 16(12): 834–7.

[27] Xiao SY (1994). Theoretical basis and research application of the social support revalued scale [J]. J Clin Psychol Med, 2: 98–100.

[28] Zhi FY (2012). On the features of psychological stress and its relationship with social comparison among ethnic minorities in southwest China. J Southwest Univ, 34(10): 150–6.

[29] Li W (2008). Association studies of mental health and coping style in postgraduates of agriculture and Forestry University. J Clin Psychosom Dis, 14(4): 339–41.

